# Prostate Tissue Microbiome in Patients with Prostate Cancer: A Systematic Review

**DOI:** 10.3390/cancers16081549

**Published:** 2024-04-18

**Authors:** Daniela F. Ward Grados, Onuralp Ergun, Carly D. Miller, Petr Gaburak, Nana A. Frimpong, Oluwatobi Shittu, Christopher A. Warlick

**Affiliations:** Department of Urology, University of Minnesota, Minneapolis, MN 55455, USA; oergun@umn.edu (O.E.);

**Keywords:** prostate cancer, prostate microbiome, *Cutibacterium*, microbiota

## Abstract

**Simple Summary:**

Prostate cancer is a leading cause of death among men worldwide. Some researchers have speculated that the prostatic microbiome is involved in prostatic inflammation and the pathogenesis of prostate cancer; however, there has not been consensus regarding specific organisms or their overall impact on this process. In order to synthesize the data that exists on the prostate microbiome, we performed a systematic review of the literature. In this review, we concluded that the methods that have been used to identify microbes within the prostate are highly variable and therefore do not constitute robust evidence. Further research is necessary to refine the methods used and to better understand which organisms may play a role in the development of prostate cancer.

**Abstract:**

Some researchers have speculated that the prostatic microbiome is involved in the development of prostate cancer (PCa) but there is no consensus on certain microbiota in the prostatic tissue of PCa vs. healthy controls. This systematic review aims to investigate and compare the microbiome of PCa and healthy tissue to determine the microbial association with the pathogenesis of PCa. We searched MEDLINE, Embase, and Scopus databases. Articles were screened by two independent and blinded reviewers. Literature that compared the prostatic tissue microbiome of patients with PCa with benign controls was included. We found that PCa may be associated with increased *Propionibacterium acnes*, the herpesviridae and *papillomaviridae* families, and *Mycoplasma genitalium*, but definitive conclusions cannot be drawn from the existing data. Challenges include the difficulty of obtaining uncontaminated tissue samples and securing tissue from healthy controls. As a result, methods are varied with many studies using cancerous and “healthy” tissue from the same prostate. The organisms chosen for each study were also highly variable, making it difficult to compare studies. These issues have led to lower confidence in our results. Overall, further work is warranted to better understand the implications of the prostatic microbiome in the pathogenesis of PCa.

## 1. Introduction

Prostate cancer continues to be the second leading cause of cancer-related deaths among men in the United States, with current statistics reporting that 1 in every 44 men will die of prostate cancer and 1 in every 8 men will develop prostate cancer in their lifetime [1]. Our understanding of prostate cancer has evolved over the years, showing the connection between many risk factors not limited to age, ethnicity, family history, and diet [2]. Current treatment options, according to the latest guidelines, include radical prostatectomy, radiotherapy, and hormone therapy as standard treatment options for patients presenting with local or regional disease, resulting in 5-year relative survival rates of greater than 99% in local and regional disease [3]. Despite the high rates of disease control with current treatment options, recurrence and disease progression to metastatic prostate cancer continue to pose challenges to patients as well as physicians [4]. To shed light on the etiology, multiple factors are being actively investigated for potential drivers of cancer progression. Prostate cancer carcinogenesis and disease progression are complex processes with numerous intrinsic and environmental factors substantially affecting these processes, including diet, obesity, inherited genetic risk alleles, smoking, social determinants of health, inflammation, and possibly infectious agents, among others [2,5,6].

More recently, the human microbiome has become an area of increasing interest owing to its potential role in various disease processes, specifically in cancers [7,8,9,10,11]. The microbiome consists of bacterial, viral, and fungal taxa that are often associated with health and wellness. However, with the advances in technology, negative impacts on the body have also been discovered in relation to the human microbiome. The major underlying mechanism for such impacts is believed to be caused by inflammation and alterations to host immunity [12]. One of the most studied areas is the gut microbiome, which has been shown to play a role in digestive tract pathologies such as inflammatory bowel disease and colorectal cancer as well as many other diseases like Alzheimer’s disease and rheumatoid arthritis [13,14,15]. The gut microbiota has also been linked to prostate cancer progression and treatment response. by metabolizing chemotherapeutic drugs and influencing resistance development [16,17,18]. This could be due to various reasons, including the gut microbiome’s ability to metabolize chemotherapeutic drugs through biotransformation, like gemcitabine, which has showed diminished effectivity when metabolized by *Mycoplasma hyorhinis* [19]. The gut microbiome also plays a role in modifying the immune function, which can lead to variation of the response and efficacy of popular immunological therapies like checkpoint inhibitors [13,20,21]. It is speculated to be caused by alterations in the intestinal microbiome causing an increased abundance of bacteria that have androgenic functions. Recent studies have found specific microbial species and described their suspected mechanism for contributing to the aforementioned effects. *Ruminococcus* spp. has been found to be enriched in patients with castration resistant prostate cancer [20,22]. This is thought to be due to the ability of this bacterium to synthesize dehydroepiandrosterone (DHEA) from pregnenolone. Thus aiding tumor growth even in an androgen-deprived environment [22]. Similarly, several studies have described the increased abundance of specific bacterial strains, such as *Akkermansia muciniphila* in the gut of patients with prostate cancer that receive treatment with androgen deprivation therapy (ADT), hence it is theorized that this bacterium might promote the antitumor effects of ADT [22,23,24]. This data proves that the microbiome can have significant effects on disease progression aside from providing new therapeutic targets. However, it is important to explore the effects that the microbiota of other areas like the urinary tract and prostatic tissue might have on this disease. Especially because these areas are more closely related to the affected organ, the prostate. Because of this, a similar link has been investigated between the genitourinary microbiome in men with prostatitis and prostate cancer. The most commonly found organisms in the urinary microbiome of patients with prostate cancer are proinflammatory organisms, including *Cutibacterium*, *Streptococcus anginosus* and *Escherichia coli* [19]. The pathogenesis of prostate cancer progression, in this case, is theorized to be the result of microorganisms producing reactive oxygen species, resulting in local inflammation that ultimately drives the carcinogenic process [25]. Previous studies have found ties between the microbiome of prostatic tissue, seminal fluid, and urine and various pathological processes such as prostatitis [26], and some studies have found a possible link between prostatitis and an increased risk of the development of cancer [27]. Furthermore, to our knowledge, there is no consensus on significant microbiota differences found in prostate cancer compared to healthy prostatic tissue controls that may be directly associated with carcinogenesis or disease progression despite tantalizing individual studies.

Understanding the mechanisms by which the microbiome influences prostate cancer is crucial in developing targeted therapeutic strategies. The microbiome has been shown to affect the local immune response within the prostate gland, with dysbiosis facilitating local chronic inflammation and potentially promoting tumor growth [7,25,28]. Exploiting these interactions may offer novel therapeutic approaches, including the exploration of interventions like probiotics, prebiotics, postbiotics and microbiota transplantation, with promising potential to prevent disease progression, alleviate chemotherapy side effects, and personalize treatment plans [29,30]. In addition to these new therapeutic options, leveraging the relationship between the microbiome and local immune processes might increase the efficacy of immunotherapies for prostate cancer such as immune checkpoint inhibitors (like anti PD-1 agents), Sipuleucel-T, and CAR-T therapy [31].

Deciphering the unique “fingerprint” of the prostatic microbiome and its interplay with prostate cancer is crucial for this personalized approach. This systematic review delves into a vital comparison, the microbial composition of prostate cancer versus noncancerous prostatic tissue, to shed light on specific microorganisms that may be involved in prostate carcinogenesis or impact disease progression or treatment response as potential areas of future medical intervention.

## 2. Methods

This review was conducted according to a PROSPERO-published protocol (CRD42023438939). In the reporting of our findings, some deviations from the protocol occurred, as the review team found no meta-analyzable data. For this reason, we instead qualitatively assessed the data and reported the species that were correlated and inversely correlated with prostate cancer development in multiple studies.

**Figure 1:**
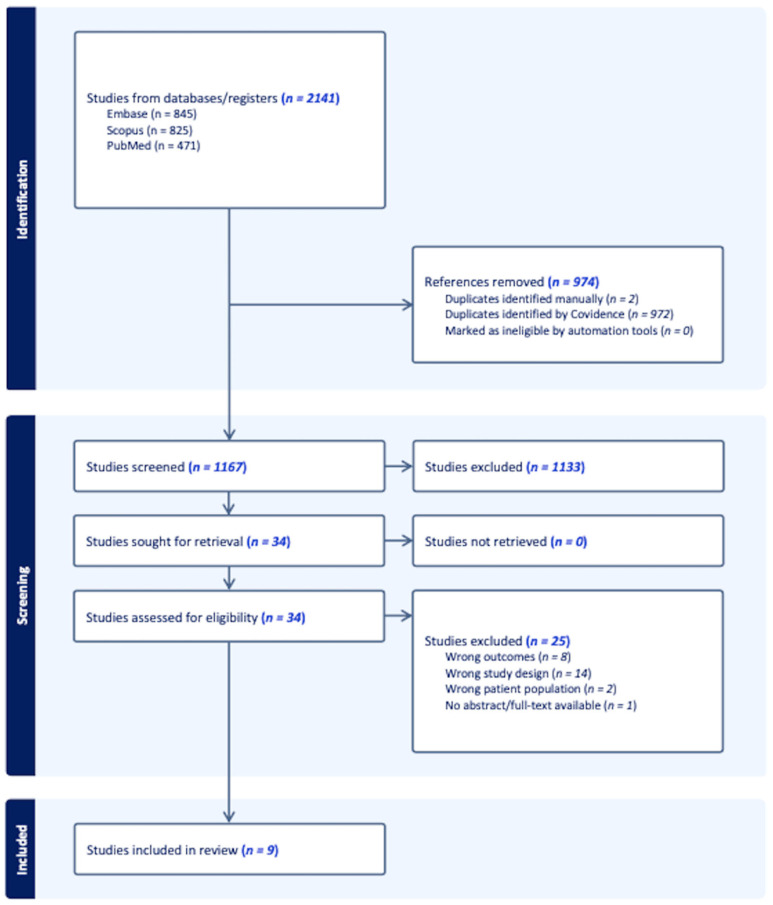
A PRISMA flow diagram.

MEDLINE, Embase, and Scopus were searched in April 2023 to capture studies relevant to the prostatic microbiome. In consultation with an expert librarian, search terms were devised and utilized to search the databases as follows: “prostate cancer” AND (microbiota OR microbiome OR metagenome OR microbial). This study was conducted by following the preferred reporting items for systematic reviews and meta-analyses (PRISMA). A tiered approach was used to review articles according to PRISMA guidelines using the Covidence platform [32]. The study screening and selection process is demonstrated in a PRISMA flow diagram (Figure 1). All authors screened articles by title and abstract to identify articles for full-text review. Each article title and abstract were reviewed by two blinded reviewers. Conflicts were then resolved by arbitration by a third reviewer. Select articles were then reviewed in full, again with two reviewers screening each text.

Studies were included if they described a comparison between the microbiome of prostatic tissue in patients with prostate cancer and that of benign control samples. We excluded studies that were case reports, case series, systematic reviews, or meta-analyses. Studies that did not specifically look at tissue samples, those that were ex-vivo studies, and those that were not human tissue were also excluded. Data from the included studies was extracted by two authors and discussed to reach a consensus. Any disagreements were discussed with the entire review team. Data included participants/samples, species found in the control group, species found in the malignant group, and whether there were subgroup analyses to stratify tumors by Gleason grade. Select studies were also discussed with an expert in the field of metagenomics to better understand study results and applicability.

## 3. Results

### 3.1. Article Retrieval

In this review, our particular interest was that of assessing the relationship that exists between the prostatic tissue microbiome and prostate cancer. In that sense, we primarily focused on defining the differences, if any, between the microbiome of benign prostatic tissue and prostate cancer. The systematic search that was conducted resulted in 2141 records, which were uploaded to the Covidence web-based platform for screening. A total of 974 duplicate records were identified and removed. During the process of title and abstract screening, 1133 articles were deemed to be irrelevant and were therefore excluded. The 34 remaining records were included for full-text review. In the end and after careful consideration, nine articles published between 2015 and 2023 were included. The remaining 24 articles were excluded due to a variety of reasons, including but not limited to: inappropriate outcome measures, inappropriate study population, or lack of full-text availability. A summary of relevant data from the included studies is presented in Table 1.

### 3.2. Diversity Assessment

Microbial diversity and the approach taken to determine it varied from article to article. Alpha diversity is a measure of the diversity or total number of organisms and their relative proportions within a defined community or space [40,42]. Beta diversity is a measurement that can differ greatly between studies as it compares the diversity of species across different samples or communities [42]. Only five out of the nine included articles, mention performing a diversity analysis. Out of those five articles, Sarkar et al., Salachan et al., and Gonçalves et al. reported that their alpha diversity assessment indicated a lower species richness in samples from the malignant group than those in the benign (control) groups [38,40,41]. However, Feng et al. and Cavarretta et al. were not able to distinguish between benign and malignant groups with alpha or beta diversity [35,37]. Four out of the five included articles did not include or mention diversity analysis. Refer to Table 1 for a summary of the diversity assessment findings of the included studies.

### 3.3. Study Outcomes

Across the nine included studies in this analysis, a total of 22 genera were found to be significantly increased in patients with prostate cancer groups compared to benign controls. Four out of the nine included studies obtained both their prostate cancer tissue samples and their non-prostate cancer controls (benign tissue) from radical prostatectomies of adult males with a clinically localized prostate cancer diagnosis [33,35,37,40]. Two studies obtained their prostate cancer tissue samples from patients who underwent radical prostatectomy for prostate cancer. In contrast, they obtained the benign tissue controls from patients with benign prostatic hyperplasia (BPH) who underwent transurethral resection of the prostate [34,39]. Two studies used transrectal ultrasound-guided prostate biopsies to obtain tissue samples for both their benign control and experimental groups [38,41]. Only one study used mRNA-seq data downloaded from EBI ENA [36].

At the genus level, *Rickettsia* [34], *Mycobacterium* [34], *Bordetella* [34], *Mycoplasma* [34,39], *Sphingomonas* [34], *Bartonella* [34], *Helicobacter* [34], *Bacillus* [34], *Porphyromonas* [34], *Salmonella* [34], *Aeromonas* [34], *Brevundimonas* [34], *Shigella* [34], *Staphylococcus* [35], *Streptococcus* [35], *Cutibacterium* [36,38,41], *Lawsonella* [38], *Shewanella* [40], *Prevotella* [41], *Cupriavidus* [41], and *Methylobacterium* [41] were found to be significantly increased in the prostate cancer cohort in several of the studies that were analyzed. However, in two studies, *Prevotella* and *Staphylococcus* were reported to be significantly more abundant in the benign groups versus the prostate cancer groups [38,40]. The other genera found to be particularly increased in the benign cohorts were *Cellvibrio* [41], *Kocuria* [41], *Vibrio* [40], *Bacteroides* [40], *Chlamydia* [34], *Pseudomonas* [34], *Burkholderia* [34], *Campylobacter* [34]. Of note, *Pseudomonas* was identified in four of the nine included studies [34,37,38,41]. In two of these studies, *Pseudomonas* was found to be present in both the benign and malignant cohorts [37,38]. In the remaining two studies, *Pseudomonas* was reported to be present exclusively in the benign cohorts [34,41]. It was significantly increased in the benign group in the Banerjee study [34]. Gonçalves et al. found that *Pseudomonas* was the most prevalent genus in the benign group, although this finding was not reported as statistically significant [38]. Aside from bacteria, four out of the nine articles also investigated the presence of viral organisms in their samples. At the family level, Banerjee et al. found Poxviridae, Reoviridae, Papillomaviridae, and Herpesviridae to have a high hybridization signal in the cancer cohort versus in their control population [34]. EBV, HBV, HPV16, and HPV18 were also found to be significantly increased in the malignant cohort in the study by Sarkar et al. [41]. Finally, *Saimiriine betaherpesvirus* was exclusively found in the benign cohort by Salachan et al. [40]. Only Banerjee et al. explored other types of organisms, including viruses, parasites and fungi [34]. Of note, this study defined results as significant if they had a log2 fold change > 1 and an adjusted *p* value < 0.05. They further classified these significant results into High, Mid, and Low depending on the size of their hybridization signal. For the purpose of our study, we only considered those with a high hybridization signal (Log2 ≥ 10) as significant for our analysis.

### 3.4. Quality Assessment

**Figure 2:**
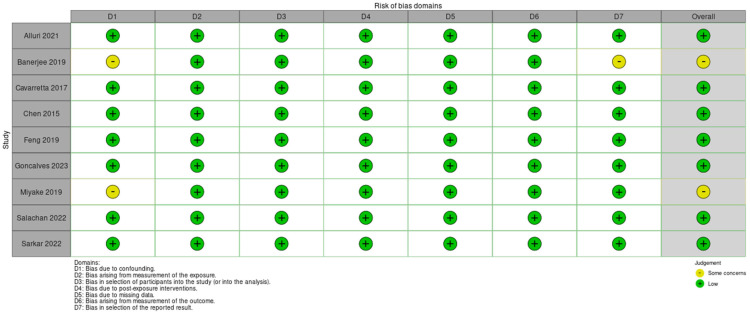
Risk of bias domains [33,34,35,36,37,38,39,40,41].

Authors working in pairs assessed the risk of bias independently. Discrepancies were resolved by discussion or arbitration with a third author. Although the authors’ judgments were favorable in most of the risk of bias domains, our confidence in the evidence in the literature remains low due to the nature of the studies included in this review supported by the fact that they were all non-randomized and retrospective studies. These articles varied greatly in their methodology in terms of what was used for sample and control cohorts, as well as which types of organisms were of interest. We followed guidance from the Cochrane collaboration on how to assess individual study level risk of bias and utilized the “risk of bias in non-randomized studies of exposures” [43]. Each study was evaluated for seven domains of potential risk of bias and each one was categorized as low, some concerns, high risk, and very high risk of bias. The highest concern of risk of bias in any domain constituted the overall risk of bias for each study. A summary of the judgment for these domains and the overall risk of bias is depicted in Figure 2.

Utilizing the Cochrane guidance for risk of bias, we deemed most of the included studies low risk of bias for their good account for major known confounders, their well-defined patient cohorts, their consistently classified exposures across study populations, their handling of missing data, their measurement methods to eliminate assessor effects, and their systematic reporting of their results even in cases when there were no significant findings.

## 4. Discussion

In recent years, the effect of the microbiome on cancer pathobiology has led to more focused work regarding the relationship between the genitourinary microbiome and prostate cancer. While our review highlights several relevant studies regarding the prostate microbiome specifically, it also emphasizes the need for additional prostate tissue microbiome studies to increase the power and quality of these findings. Studies to date focus on exploring bacterial and viral signatures in prostate cancer tissue in a broad manner.

It is known that the male genitourinary tract is commonly colonized by *Corynebacterium*, *Streptococcus*, *Staphylococcus*, *Finegoldia*, *Peptoniphilus*, *Anaerococcus*, and *Lactobicillus* [38]. The bacterial taxa that were found to be significantly increased in prostate cancer tissue amongst the nine studies had varied results with minimal overlap. *Propionibacterium acnes* (*P. acnes*) was the only bacterium found to be commonly increased in prostatic tissue in three of the nine studies. Several other studies have demonstrated significant expression of *P. acnes* in prostate cancer and prostatitis in human and rodent cell lines [44,45]. The pathophysiology of prostate cancer progression from *P. acnes* inoculation is theorized to be due to a chronic inflammatory state as a result of increased production of proinflammatory cytokines and neutrophil recruitment [46,47]. Furthermore, *P. acnes* has been proposed to induce cancer progression through an immunosuppressive environment as a result of macrophage gene alterations causing increased recruitment of T regulatory cells [48,49]. A study conducted by Yow et al. found that *cutibacterium* (previously known as *propionibacterium*) was also significantly increased in aggressive prostate cancer tissue compared to the healthy control [50]. Conversely, previous literature including some of the cited studies reported *P. acnes* to be prevalent in both benign and malignant prostate tissue [45,51]. Therefore, further explorations of the bacterial taxa are warranted to determine their significance in the progression of prostate cancer.

In our review, the viral taxa were only investigated in four out of the nine studies. Most commonly found were the *Herpesviridae* and *Papillomaviridae* (HPV) strains. HPV is a well-studied virus that has been shown to be associated with numerous malignant processes including cervical, penile, anal, vaginal, and various head and neck cancers [52]. A commonly proposed mechanism in cancer development via HPV is related to the virus’s ability to inactivate tumor suppressor genes p53 and pRb via the E6 and E7 oncoproteins [53]. There have been several studies that explored an association between oncogenic viruses and prostate cancer, with only some being able to report a clear association with HPV and others showing mixed results as the virus was found in both prostatic cancer and benign tissue [54,55,56,57,58]. It has been speculated that these varied results are the result of differing methodologies, specifically in the collection, primers, and assays used for viral detection. Nonetheless, this emphasizes the need for a standard methodology when examining microbiomes in relation to human cancer cells, to have a clearer understanding of viral associations in the progression of prostate cancer.

*Herpesviridae* was also found to be increased in two of the four studies that looked at viruses in our review with Epstein–Barr virus (EBV) being one of the commonly found strains [40,41]. EBV has been extensively studied and has been shown, in addition to HPV, to be one of the larger contributors to virus-associated cancers [59]. Generally, these viruses are commonly found in benign tissue as a result of sexual transmission but are reported to be the cause of several carcinomas [60,61,62]. EBV was found to have carcinogenic potential by inhibiting apoptosis and stimulating cell survival via the EBV nuclear antigen 1 and latent membrane protein 1 [63,64]. While the direct association of EBV to PCa has not been elucidated, there has been literature that showed co-inoculation of prostate tissue with HPV to be associated with PCa progression [54,65]. A study performed by de Lima et al. examined HPV and EBV co-inoculation of cervical carcinoma, and proposed that EBV infection accelerated the integration of HPV genomes into the normal cell genomes [66]. This has further been shown in a study by Nahand et al. that reported maximum integration of the HPV genome when coinfected by EBV compared to the control [65]. Therefore, the assumption is that the viruses act simultaneously to increase cell proliferation and inhibit cell apoptosis via different mechanisms.

Future studies may consider focusing on these specific bacterial and viral families to validate their association with prostate cancer in addition to exploring other novel microorganisms. It is also necessary to point out that only when the literature reaches a consensus on which microorganisms form the benign prostate microbiome, will we be able to elucidate the full extent and consequences of the modifications that it suffers during the development of cancer.

Similarly, the reviewed works call attention to the need for standardized outcome reporting when assessing biodiversity and microorganism abundance in the prostate cancer tissue microbiome. Of the studies we reviewed, each one utilized a different outcome measure, including but not limited to bacterial reads per human genome reads, percent RNA reads matched to the human genome, percent abundance, CT values for bacterial load/abundance, percent of samples with specific organismal DNA, and hybridization signal intensity. The lack of standardization and consensus in methodology for reporting these outcomes further complicates our ability to compare results and draw conclusions from the existing literature. Based on the microbiome work available to date, future studies should focus on reporting outcomes that are standardized and reproducible to best characterize the prostate cancer microbiome in an easily understandable, comparable, and potentially clinically relevant manner. We also need to consider the particular limitations of the detection techniques when analyzing microbiome research. Amplicon sequencing is currently one of the most used techniques of next generation sequencing, it relies on PCR amplification of the bacterial 16s ribosomal RNA, which contains nine hypervariable regions. However, only a subset of these hypervariable regions is targeted in most studies for cost reduction. This action can result in bias introduction and inaccurate representation of the abundance of the studied bacteria. In a similar manner, DNA/RNA extraction methods, the sequencing platform used, and even storage conditions may add variability to the results. Because of this, the same technique needs to be used for all samples in the study so as to avoid unnecessary confounders during analysis. Each one of these aspects needs to be clearly stated in the manuscripts for transparency and clarity [67].

The current standard for “healthy or non-cancerous” prostate tissue and prostate cancer tissue should be examined as well. Most “healthy” prostate tissue samples that were utilized as benign controls were taken from a region of the prostate without cancerous cells from the prostate glands of patients diagnosed with prostate cancer. This method not only risks missing key microbiota changes that may occur in precancerous tissue but may also confound the data with an individual’s unique microbiome. Although difficult to obtain, prostate tissue from healthy controls without prostate cancer, rather than cancerous prostate tissue, would be ideal to identify the differences in the microbiome. The best example of this is the techniques used by some of the included studies, which utilized benign tissue samples from patients with benign prostatic hyperplasia who underwent transrectal resection of the prostate.

Although the limitations outlined above led to lower quality data in this review, the studies we reviewed and included have provided a more focused microbiome to be explored in future studies. Additionally, as this area of research is fairly new, the literature reviewed yields important insight into the best way to structure novel studies and report findings in a significant and uniform way.

## 5. Conclusions

With the current evidence available, we are able to elucidate a likely relationship between microorganisms, like *Cutibacterium* and Herpesviridae, and PCa, as they seem to be increased in this population. However, there are major limitations to the current research, which makes it difficult to generalize or draw extensive conclusions about these relationships. Further research is needed to test these associations by focusing on implementing a standard approach to outcomes reporting and obtaining benign prostatic tissue from healthy patients to serve as an optimal control group.

## Figures and Tables

**Table 1:** Summary of microbiome detection.

Study ID	Detection Method/Data Extraction Kit	Number of Participants/Mean Age (Years)	Sample Type	Detected Organisms				Diversity
				Bacteria	Virus	Fungi	Parasites	
AlluriLSC, 2021 [33]	qPCR/QIA amp DNA mini kit (Qiagen USA)	30 participants/51 to 74 years	Exp: Areas of cancerous tissue identified by pathologists from samples obtained from radical prostatectomies of adult males.	*F. nucleatum* (No significant difference [folder change] among benign and cancerous tissue)	-	-	-	Not mentioned
			Control: Areas of BPH tissue identified by pathologists from samples obtained from radical prostatectomies of adult males.	*F. nucleatum* (No significant difference [folder change] among benign and cancerous tissue)	-	-	-	
Banerjee, 2019 [34]	PCR/TransPlex Complete Whole Transcriptome Amplification Kit	50 samples/not mentioned	Exp: prostate adenocarcinoma samples from patients who underwent prostatectomy.	***Rickettsia***, ***Mycobacterium***, ***Bordetella***, ***Mycoplasma***, ***Sphingomonas***, ***Bartonella***, ***Helicobacter***, ***Bacillus***, ***Porphyromonas***, ***Salmonella***, ***Aeromonas***, ***Brevundimonas***, ***Shigella***.	***Poxviridae***, ***Reoviridae***, ***Papillomaviridae***, ***Herpesviridae***;	***Alternaria***, ***Malassezia***, ***Candida***, ***Cladosporium***, ***Trichosporon***, ***Cladophialophora***, ***Rhodotorula***, ***Geotrichum***, ***Fusarium***, ***Nosema***, ***Mucor***, ***Pleistophora***.;	***Plasmodium***, ***Trichinella***, ***Sarcocystis***, ***Babesia***, ***Entamoeba***.	Not mentioned
		15 samples/not mentioned	Control: BPH samples obtained from patients who underwent TURP	***Chlamydia***, ***Pseudomonas***, ***Burkholderia***, ***Campylobacter***.	***Retroviridae***, ***Poxviridae***, ***Reoviridae and Herpesviridae***	*Candida*, *Absidia*, *Filobasidiella*, *Cunninghamella*, *Nosema*, *Curvibasidium*, *Histoplasma*, *Encephalitozoon*.	***Babesia***	
Cavarretta, 2017 [35]	16S sequencing of variable regions 3 to 5/QIAamp DNA FFPE Tissue Kit	16 participants/65 years	Exp: Areas of cancerous tissue were identified by pathologists from samples obtained from radical prostatectomies of adult males.	*Propionibacterium*, *Corynebacterium*, ***Staphylococcus***, *Gemellales*, *Paracoccus*, *Micrococcus*, ***Streptococcus***.	-	-	-	Beta Diversity could not differentiate between the cohorts.
			Control: Areas of non-tumoral tissue were identified by pathologists from samples obtained from radical prostatectomies of adult males.	*Propionibacterium*, *Corynebacterium*, *Streptococcus*, *Staphylococcus*, *Gemellales*, *Paracoccus*, *Micrococcus* (None of these genera were found to be significantly increased with respect to the cancerous tissue samples)	-	-	-	
Chen, 2015 [36]	16S rRNA sequencing/Ribopure kit (Ambion)	20 prostate tumor samples/not mentioned	Exp: The mRNA-seq data were downloaded from EBI ENA	***Propionibacterium acnes***	-	-	-	Not mentioned
		10 matched adjacent tissues/not mentioned	Control: The mRNA-seq data were downloaded from EBI ENA	-	-	-	-	
Feng, 2019 [37]	PCR/QIAquick PCR Purification Kit (Qiagen, Germany)	65 participants/68.4 ± 7.3 years	Treatment-naïve prostate tumour and matched benign tissue were collected from the radical prostatectomy series at the Shangai Changai Hospital	Metagenome: *Escherichia*, *Propionibacterium*, *Acintobacter*, *Staphylococus*, *Pseudomonas*, *Ralstonia*, *Bacteroides*, *Streptococcus*, *Enterobacter*, *Bacillus.*; Metatranscriptome: *Pseudomonas*, *Escherichia*, *Acintobacter Propionibacterium*, *Serratia*, *Klebsiella*, *Delfita*, *Ralstonia*, *Staphlococcus*, *Morxella*	-	-	-	Alpha Diversity could not distinguish between benign and malignant cohorts. Beta diversity demonstrated that paired samples from the same patient were more similar in bacterial composition to each other than between different patients. NMDS could not differentiate between malignant and benign cohorts.
Gonçalves, M.F.M., 2023 [38]	16S rRNA sequencing/QIAamp DNA Micro Kit	15 participants/68 ± 9 years	Prostate biopsy specimens were obtained from adult males. Negative biopsies were used as controls.	Tax Comp: *Pseudomonas*, *Faecalibacterium*, ***Cutibacterium***, *Bacteroides*, *Corynebacterium*, *Turicella*, *Curvibacter*, *Sphingomonas*, *and Staphylococcus.* Bacterial Core Community: not-assigned genus, followed by *Pseudomonas*, *Cutibacterium*, *Curvibacter*, *Sphingomonas*, *Corynebacterium*, *Staphylococcus*, ***Lawsonella***, *and Paracoccus.* Differential Abundance of Bacterial Taxa: *Alishewanella*, *Paracoccus*, *Klebsiella*, *and Rothia*	-	-	-	Alpha Diversity did not show significant differences between cohorts, but species richness was lower in PCa patients than in the non-PCa group. Beta diversity showed no differences between PCa and non-PCa groups.
		15 participants/ 69 ± 8 years		Tax Composition: ***Prevotella***; Bacterial Core Community: *Pseudomonas* was the most prevalent genus in non-PCa patients, followed by a not-assigned genus, *Sphingomonas*, *Curvibacter*, *Corynebacterium*, and *Cutibacterium*. Differential abundance of Bacterial Taxa: *Actinomyces*, *Parabacteroides*, *Prevotella*, and members of the family *Muribaculaceae*	-	-	-	
Miyake, 2019 [39]	PCR/NucleoSpin^®^ DNA FFPE XS	45/67.5 years	Exp: Prostatic tumor tissue was optained trhough RALP	***M. genitalium***	HPV18, HPV16	-	-	Not mentioned
		33/71.4 years	Control: Benign prostatic tissue was obtained through TURP of patients with BPH	*M. genitalium*	HPV18	-	-	
Salachan, 2022 [40]	Metatranscriptomics/RNeasy Plus Mini Kit (Qiagen)	94 patients/65.7 years	Both benign and malignant tissue samples were obtained by curatively intended radical prostatectomy of patients with histologically confirmed localized prostate cancer.	***Shewanella***, *Bacteroides fragilis*, *saimiriine betaherpesvirus*, *vibrio parahaemolyticus*, *staph saprophyticus*	-	-	-	Alpha diversity analysis was accomplished by using Observed, Chao1, ACE, Shannon, Simpson and Inverse Simpson. These measures showed an overall reduction in species diversity in malignant compared to benign cohorts.
				***Staph saprophyticus***, ***Vibrio parahaemolyticus*** and ***Bacteroides fragilis***	**Saimiriine betaherpesvirus**	-	-	
Sarkar, 2022 [41]	16s rRNA sequencing/Not mentioned	33 PCa samples/65.1 years	Both BPH and Malignant tissue samples were obtained by TRUS-guided biopsy of patients with PSA > 4 and no prior therapy.	***Prevotella copri***, ***Cupriavidus campinensis***, ***Propionibacterium acnes***, ***Cupriavidus taiwanensis***, ***Methylobacterium organophilum***, *Brevundimonas vancanneytii*, *Neisseria flavescens*, *Acinetobacter junii*, *Bradyrhizobium cytisi*, *Cupriavidus basilensis*, *Caulobacter segnis*, *Leclercia adecarboxylata*, *Neisseria elongata*.	**EBV**, **HBV**, **HPV16**, **HPV18**	-	-	Alpha diversity showed a significantly decreased richness in the malignant cohort compared to the benign cohort. Beta diversity did not show any significant differences between the groups.
		16 BPH samples/64.8 years		***Kocuria palstris***, ***Cellvibrio mixtus***, *pseudomonas stutzeri*, *paracoccus*, *staph hominis*, *corynebacterium tuberculosteari*, *brachybacterium paraconglomera*, *staph arlettae*, *staph cohinii*, *anaerococcus octavius*	-	-	-	

Results marked in bold represent statistically significant findings. Exp: Experimental group (prostate cancer tissue), Control: Control group (benign tissue), BPH: Benign prostatic hyperplasia, TURP: Transurethral resection of the prostate. EBV: Epstein–Barr virus, HBV: Hepatitis B virus, HPV: Human papillomavirus, PCa: Prostate cancer.
